# Spatial modelling of malaria cases associated with environmental factors in South Sumatra, Indonesia

**DOI:** 10.1186/s12936-018-2230-8

**Published:** 2018-02-20

**Authors:** Hamzah Hasyim, Afi Nursafingi, Ubydul Haque, Doreen Montag, David A. Groneberg, Meghnath Dhimal, Ulrich Kuch, Ruth Müller

**Affiliations:** 1 Institute for Occupational Medicine, Social Medicine and Environmental Medicine, Faculty of Medicine, Goethe University, Frankfurt am Main, Germany; 2 Faculty of Public Health, Sriwijaya University, Indralaya, South Sumatra Indonesia; 3 Remote Sensing Program, Faculty of Geography, Gadjah Mada University, Yogyakarta, Indonesia; 4 Department of Public Health, Baldwin Wallace University, Berea, OH USA; 5 Barts and the London School of Medicine, Centre for Primary Care and Public Health, Queen Mary University of London, London, UK; 6 Nepal Health Research Council (NHRC), Ramshah Path, Kathmandu, Nepal

**Keywords:** Geographically weighted regression (GWR), Ordinary least squares (OLS), Akaike information criterion (AIC), Physical environment, Local climate, Sumatra, Rainfall, Elevation, Distance to water

## Abstract

**Background:**

Malaria, a parasitic infection, is a life-threatening disease in South Sumatra Province, Indonesia. This study aimed to investigate the spatial association between malaria occurrence and environmental risk factors.

**Methods:**

The number of confirmed malaria cases was analysed for the year 2013 from the routine reporting of the Provincial Health Office of South Sumatra. The cases were spread over 436 out of 1613 villages. Six potential ecological predictors of malaria cases were analysed in the different regions using ordinary least square (OLS) and geographically weighted regression (GWR). The global pattern and spatial variability of associations between malaria cases and the selected potential ecological predictors was explored.

**Results:**

The importance of different environmental and geographic parameters for malaria was shown at global and village-level in South Sumatra, Indonesia. The independent variables altitude, distance from forest, and rainfall in global OLS were significantly associated with malaria cases. However, as shown by GWR model and in line with recent reviews, the relationship between malaria and environmental factors in South Sumatra strongly varied spatially in different regions.

**Conclusions:**

A more in-depth understanding of local ecological factors influencing malaria disease as shown in present study may not only be useful for developing sustainable regional malaria control programmes, but can also benefit malaria elimination efforts at village level.

## Background

Malaria is a significant public health concern worldwide, including Indonesia [1]. The Indonesian government has set a national goal to be malaria-free by 2030. Currently, 24 out of 576 districts in Indonesia classified as being malaria endemic, and an estimated 45% of Indonesia’s total population are living at risk of contracting malaria [2]. In South Sumatra Province, the malaria incidence was 0.46 per 1000 people in 2013. In this province, the proportion of children under 5 years of age who applied mosquito nets was 32.7%, and the percentage of children under five who treated for fever with antimalarial medication was 89.8% in 2013 [2]. Malaria elimination has been a priority in the millennium development goals (MDGs) [3], and since then has continued to be central to the sustainable development goals (SDGs), supporting Indonesia’s malaria elimination commitments [4]. It is now essential to generate the knowledge that is necessary to develop lasting policies for the national malaria elimination programme.

Several meteorological and environmental variables are risk factors for malaria [5]. Since specific meteorological, environmental factors are at interplay and different factors can affect malaria transmission within a given province [3, 6, 7], it is important to differentiate between factors that influence the vector, the parasite and the host-vector relationship [8]. Atieli et al. have demonstrated that the topographic variables elevation, slope, and aspect are influencing the development of *Anopheles* mosquitoes [9]. In north-eastern Venezuela, there is a significant association of malaria transmission with local spatial variations like population density, lowland location, and proximity to aquatic environments [10]. Elsewhere (e.g., Ethiopia and Senegal) spatial relationships between climatic variability like rainfall and malaria occurrence have been demonstrated [11]. Rainfall indirectly benefits *Anopheles* mosquitoes by increasing relative humidity which prolongs adult longevity [12], and the number of breeding places which in turn favours population growth [13]. Temperature and the extent of water availability for larval breeding are crucial factors in the vector life-cycle, affecting transmission [3]. Vectors and parasites are both highly sensitive to any temperature changes, for example, the parasite proliferation depends on temperatures [14]. Temperatures above 28 °C have been shown to reduce malaria incidence in Africa [15]. In Indonesia, the optimum temperature for malaria mosquitoes ranges between 25 and 27 °C [3]. For the vector-host relationship, factors such as the distance of people’s houses from a river, lakes, pond, distance to the regional urban centre [16–18] distance to forest [19, 20] were shown to be significant predictors.

Spatial nonstationary is a condition in which a simple “global” model cannot define the relationship amongst several sets of variables [21]. Thus, global OLS and local GWR modelling was performed to analyse the environmental risk factors for malaria in South Sumatra that vary geographically at the regional level. The locally different ecological factors studied to potentially predict the response variable ‘confirmed malaria case’ (Y) are altitude (X1), aspect (X2), distance from the river (X3), distance from lakes to pond (X4), distance from the forest (X5), and rainfall (X6).

## Methods

### Study area

**Fig. 1:**
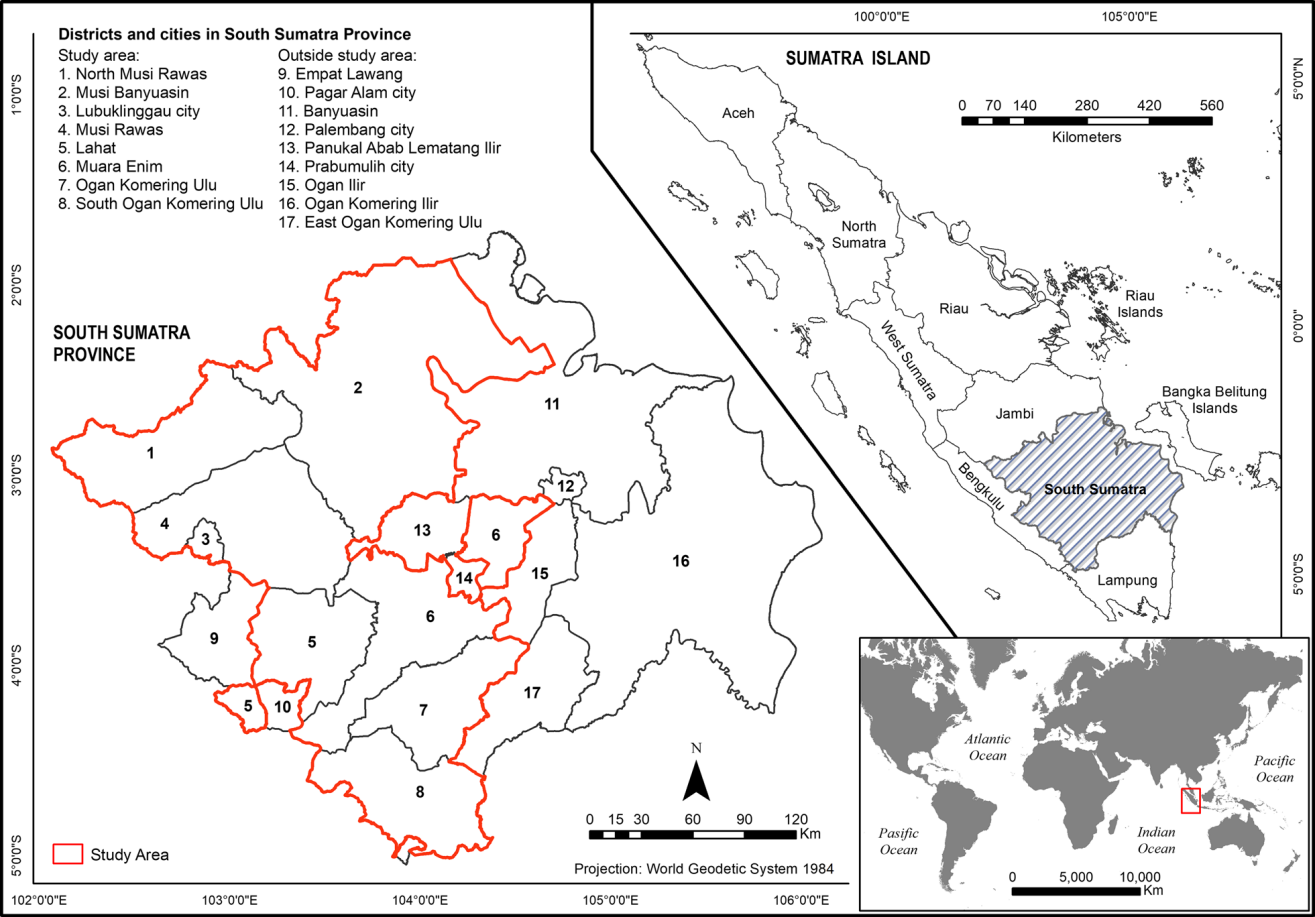
Map of the study area covering one city and seven districts of South Sumatra Province, Indonesia

The study area is located between 1°46′ and 4°55′ of southern latitude and between 102°4′ and 104°41′ of eastern longitude and has a total surface area of 46,377.40 km^2^ (Fig. 1). It covers eight endemic malaria districts of South Sumatra, Indonesia, namely Lahat, Muara Enim, Musi Banyuasin, Musi Rawas, North Musi Rawas, Ogan Komering Ulu, South Ogan Komering Ulu, and Lubuk Linggau. The topography of the area varies from lowland to mountainous landscapes. The elevation in the study area varies between 0 and 3150 metres above sea level. The climate is tropical and wet [22]. In 2013 in South Sumatra, the lowest rainfall was 31 mm (August) in Lahat district, and the highest rainfall was 613 mm (March) in Palembang City. Monthly average temperatures ranged from 26.6 to 28.3 °C and relative humidity from 81 to 88% in 2013 [23].

Indonesia’s South Sumatra Province is home to 7828,700 inhabitants. In 2013, the gross regional domestic product (GRDP) with oil and gas was IDR 231.68 trillion (17.32 billion USD) [22], based on IDR to USD exchange rates at the time of writing. South Sumatra is an ethnically highly diverse province and home to different local languages and diverse cultural and socioeconomic practices [2]. Local people engage in coffee, rubber and palm oil plantation activities or work in the industrial mining area, which shapes not only people’s lives but also the environment [24]. Indonesia contributes significantly to deforestation in Southeast Asia. Recent developments of deforestation have led to unsustainable practices which have resulted in a high frequency of deforestation in some regions and are an important factor influencing malaria incidence [25]. Deforestation has been shown to be connected with malaria incidence in the county (Município) of Mâncio Lima, Acre State, Brazil. There, a cross-sectional study shows 48% increase in malaria incidence are associated with cumulative deforestation within respective health districts in 2006 [26].

### Study population and data collection

**Fig. 2:**
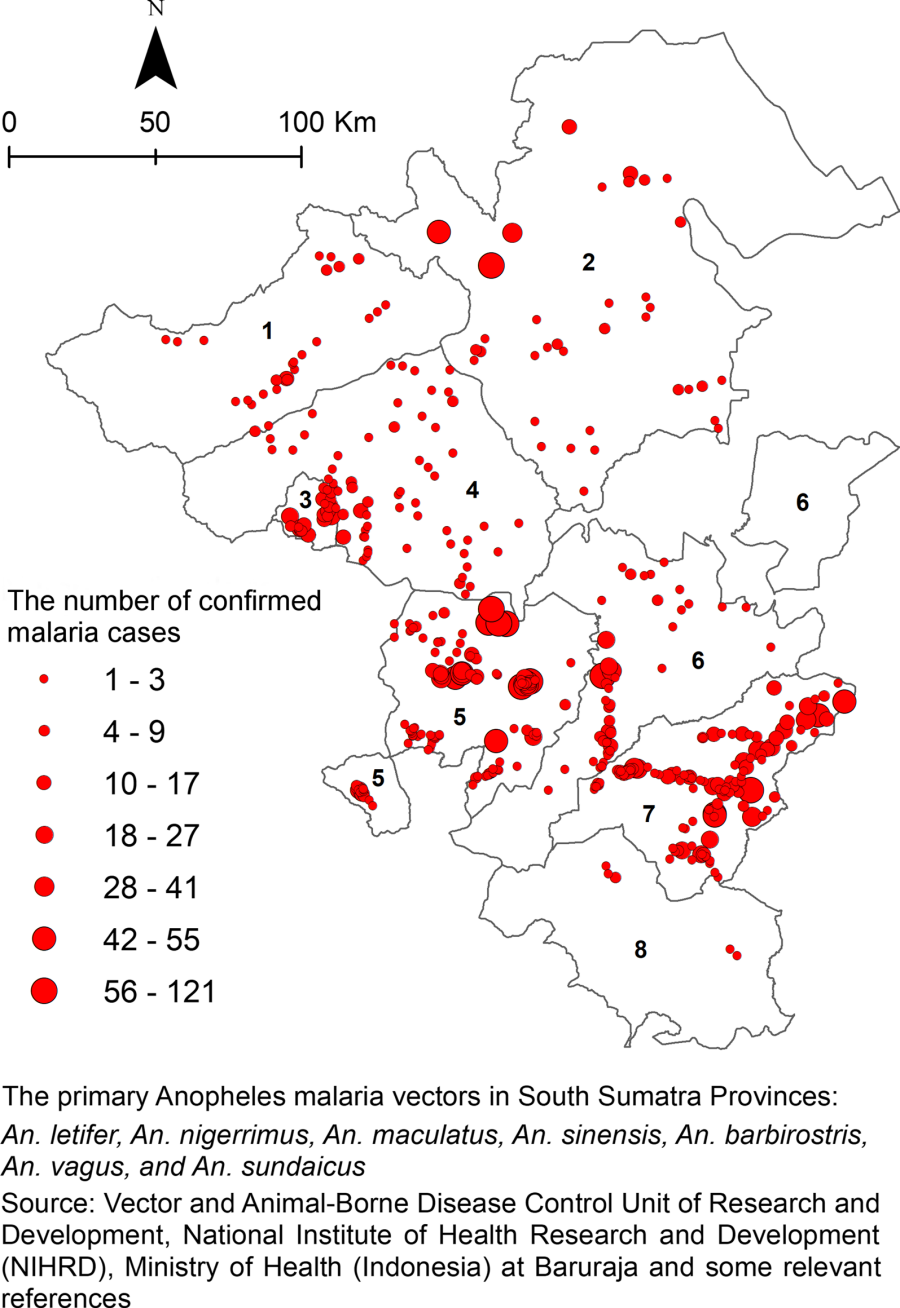
Malaria cases and their geographical locations in the study area

**Fig. 3:**
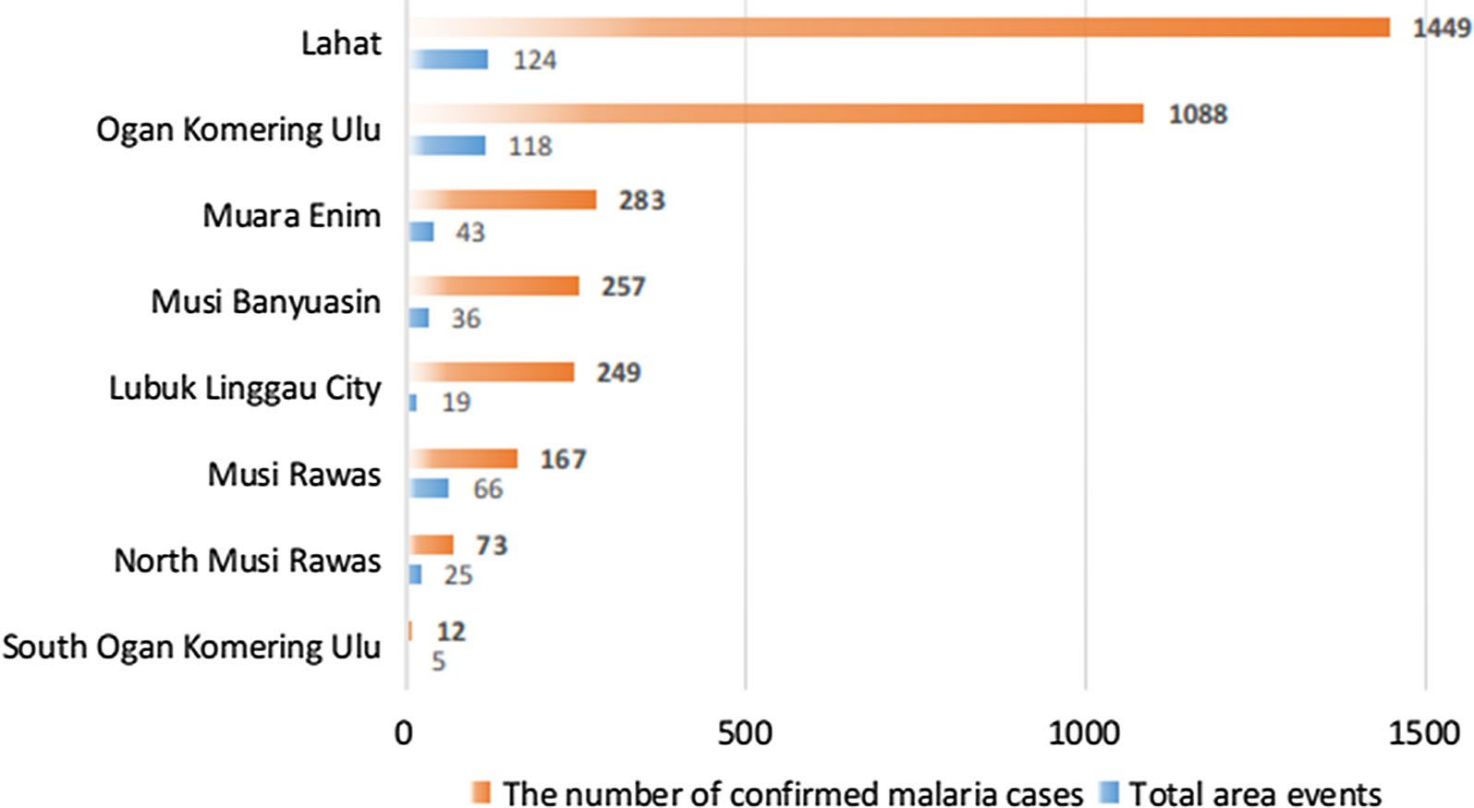
Malaria cases at village level

36,372 patients seeked treatment due to suspected malaria fever in 140 primary health centres (PHC) in the study region South Sumatra during January to December 2013. Among them, 3578 were laboratory positive for malaria. The cases spread over 436 out of 1613 villages that were used for unit analysis. The detailed number of malaria cases in different provinces are presented in Fig. 2. The spatial distribution of participants who had confirmed cases of malaria is shown in Fig. 3.

The patients are categorised into “clinical diagnosis”, “suspected malaria” and “confirmed malaria cases”. Categories “clinical diagnosis” or “suspected malaria” are based on the patient’s symptoms and physical findings at examination. A “confirmed malaria case” is a case of malaria diagnosed microscopically (examination of blood specimen/preparation) or rapid diagnosis test (RDT) with positive results for *Plasmodium*. Either RDT or microscopic assessment or both were used to confirm the diagnosis of malaria. The malaria diagnostic data were obtained from the regular health information reporting system of the Provincial Health Office of South Sumatra. The data had been collected during 12 months (January to December 2013) at the village level from patients seeking treatment in PHC, locally called Pusat Kesehatan Masyarakat (“puskesmas”), and that were reported monthly to the Provincial Health Office via the malaria programmes in the District Health Offices.

### Geographic information

**Fig. 4:**
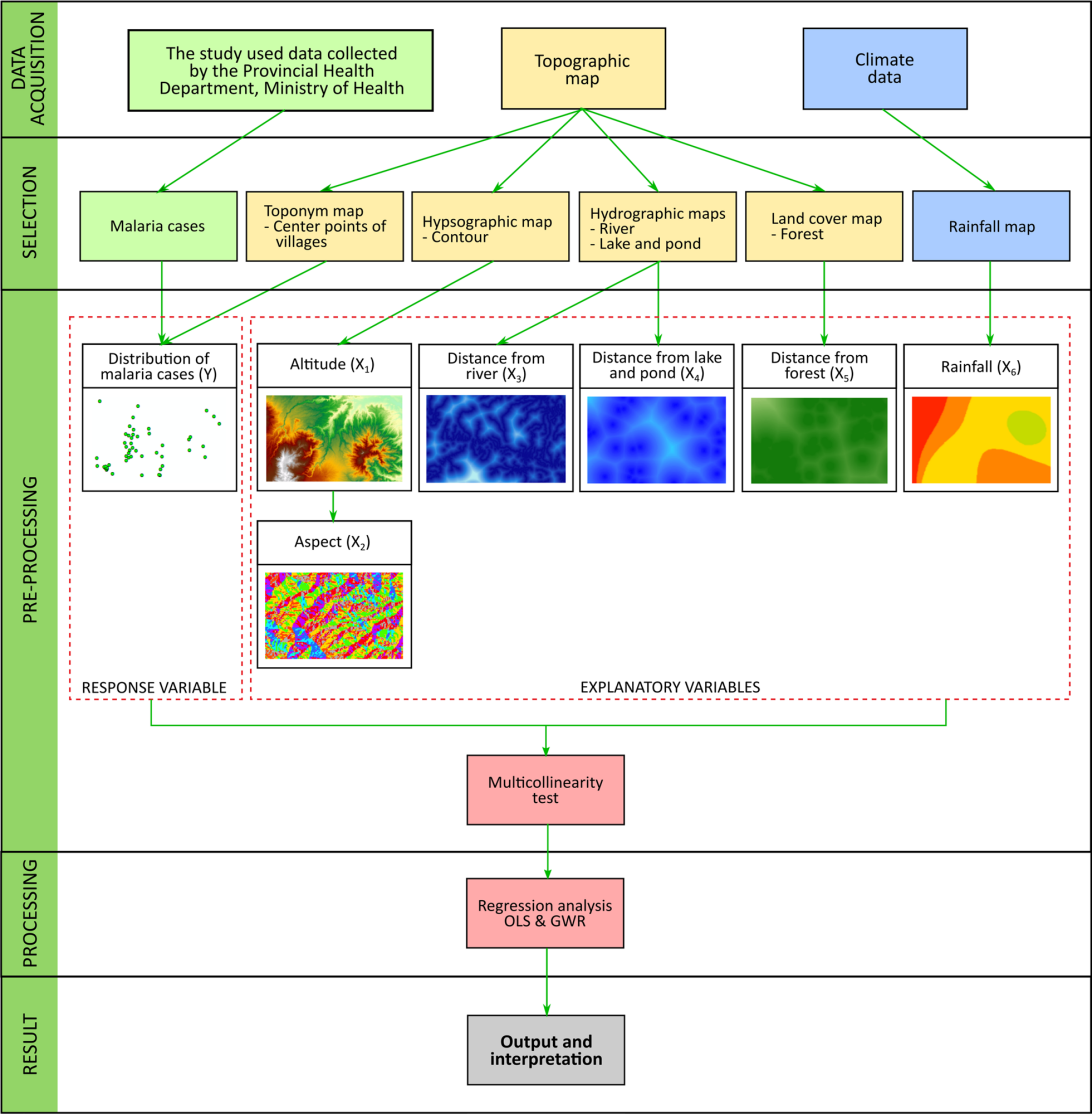
Flow chart of the research strategy

The study area map (Fig. 1) uses the World Geodetic System (WGS84) as its reference coordinate system. As shown in Fig. 4, three stages of working with geographic information were distinguished: data acquisition and processing, data analysis and data presentation [27]. GWR 4.0 version 4.0.90 and Arc GIS 10.3 were used for data processing, analysis, and visualization. Malaria case data were collected from the Provincial Health Department, Ministry of Health (see previous paragraph) as well as topographic (toponymy map, hypsographic map, hydrographic maps, land cover map) and climate data (rainfall map). The primary spatial data were obtained from a topographical map of Indonesia (cartographic material) which has a scale of 1:50,000 and consists of several layers of plots grouped. The malaria input data is aggregated village level data with the village centroid used as the spatial unit. This map consisted of a collection of geographic data presented as thematic layers for land cover, hydrographic data and a sheet of hypsography. Indonesian topographic map known as Peta Rupabumi Indonesia (RBI) was updated in 2014. In 2013, topographic data visualisation has been changed into geodatabase cartography to reduce the steps of creating cartography visualisation in topographic mapping activity [28]. These maps were obtained from the Geospatial Information Agency (BIG) of Indonesia. Authorization for the use of the topographical map of Indonesia was provided by the Indonesian Geospatial Information Agency. However, restrictions were put to use the availability of these data and therefore are not publicly available. Data were collected by creating a research protocol which is used under license for the current study. The data that backs the findings of the research are served in the main paper.

The forest cover maps were extracted from the land cover map in 2013 on the scale of 1:250.000. The map was sourced from Ministry of Environment and Forestry, Indonesia. The precipitation map (annual average) was obtained by inserting the data of average yearly rainfall from BMKG Climatological Station Class I in Palembang, South Sumatra, Indonesia. The distance between weather observation stations was 50–100 km in flat topography and 10 km in hilly terrain.

### Data pre-processing

**Fig. 5:**
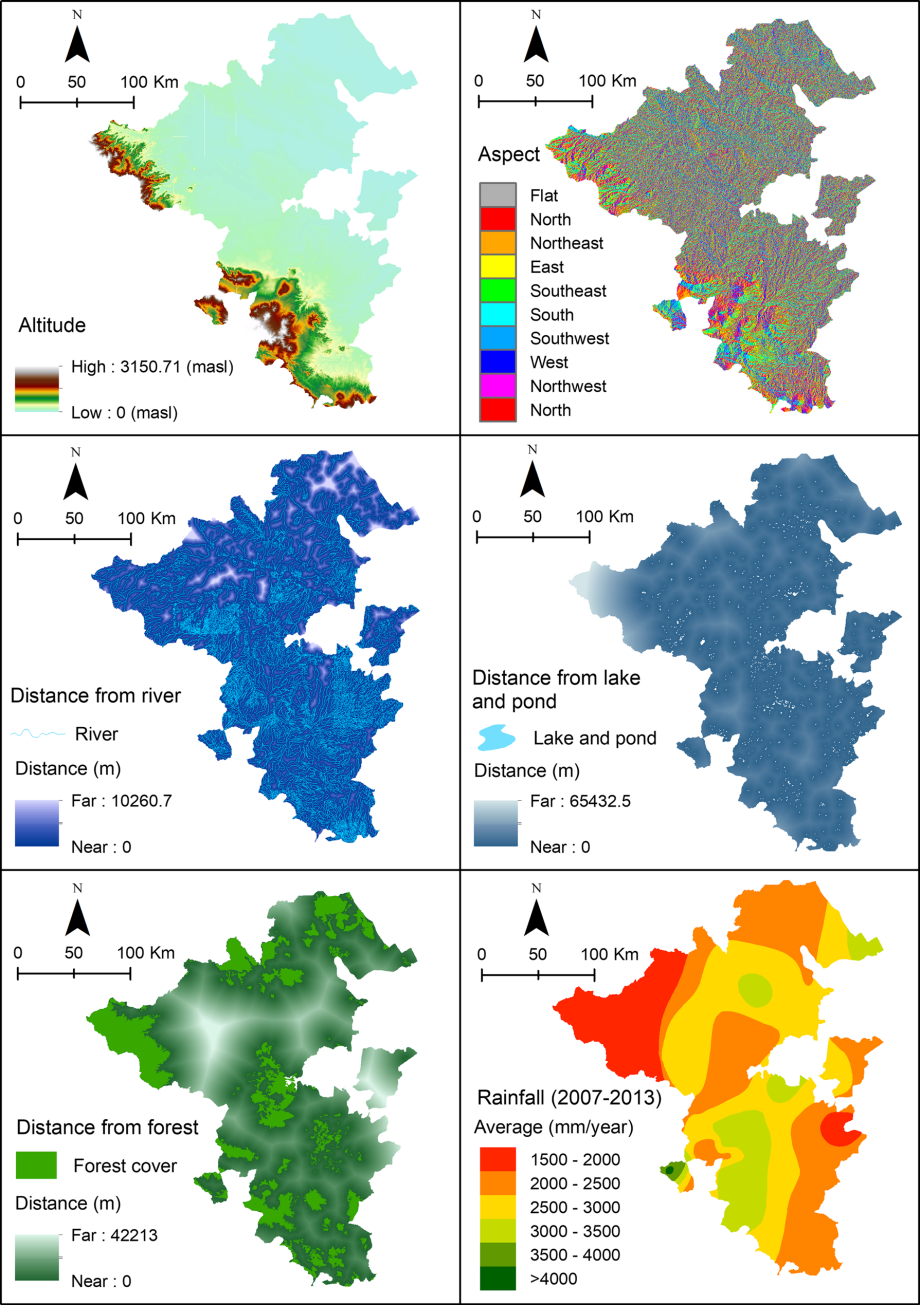
Each explanatory variable mapped in the study area

The malaria distribution map (Fig. 2) was created and six selected explanatory variables plotted (Fig. 5). The altitude map was obtained by interpolation and contouring of the map into a digital elevation model (DEM). Subsequently, the DEM data was converted into a map containing the direction of the slope (aspect). The parameter distance from the river, and distance from lake and pond processed from river, lakes, and ponds maps which were derived from the topographic map whereas distance from the forest processed from forest cover map. These variables were analysed using Euclidean distances. Rainfall parameter was calculated based on annual average rainfall over 5 years, and it was interpolated from several weather observation stations in study area. The rainfall map (isohyets map) was obtained from the scanned maps which are the result of interpolation and classified into several classes. The map needed to be rectified and digitised to get a digital rainfall map.

### Data processing and modelling

The response variable “malaria case” and explanatory variables “altitude/aspect”, “distance from river”, “distance from lake and pond”, “distance from forest” and “rainfall” were tested for multicollinearity. Therefore, the values of all explanatory variables were extracted for each case location. An index based on predictive modelling variance, the variance inflation factor (VIF) was used [29]. Multicollinearity could occur when one independent variable was a linear function of another independent variable and previously observed in GWR modelling [30]. The pattern of connection between confirmed malaria cases and environmental factors was expressed by the OLS method. Here, OLS model is called global regression model because the existence of local variation had not taken into account in regression so that the estimate of the regression remained constant. Thus, the regression parameters had the same value for each point within the study area. If spatial heterogeneity occurred in regression parameters, then the information that could not be processed by the global regression model was seen as an error. In such cases, the global regression model was less able to explain the actual data phenomenon [31]. A global regression coefficient value close to zero indicated that the explanatory variables had a small effect on the response variable.

As alternative, the GWR model was used to investigate the relationships between response and explanatory variables since the study area was characterized by spatial heterogeneity [32]. A semiparametric GWR4.09 for Windows (provided by Nakaya et al. [32]) was carried out which is a new release of the windows application software tool for modelling spatially varying relationships among variables by calibrating GWR.

The estimated parameter of the GWR model uses the least squares given the location coordinates as a weighting factor. The influence of the points in this neighbourhood varies according to the distance to the central point [33]. The optimum distance threshold (also known as the bandwidth) or the optimum number of neighbours determined in two ways: by minimising the square of the residuals cross-validation (CV) or by minimising the Akaike Information Criterion (AIC) [34]. At this stage, the type of weighing (kernel type) and optimum bandwidth selection method based were selected on AIC selection criteria. Classic AIC chooses smaller bandwidths in geographically varying coefficients are possible to be under smoothed [32]. In a GWR context, the measurement of utility is the AIC to know whether a global regression model or GWR is most useful [33].

The local GWR model as earlier described is as follows:

1 $$y_{i} = \beta_{0} \left( {u_{i} ,v_{i} } \right) + \mathop \sum \nolimits_{k} \beta_{k} \left( {u_{i} ,v_{i} } \right)x_{ik} + \varepsilon_{i}$$

Based on the model, $y_{i}$, $x_{ik}$, $\left( {u_{i} ,v_{i} } \right)$, $\beta_{k} \left( {u_{i} ,v_{i} } \right)$, and $\varepsilon_{i}$ were sequentially the response and explanatory variables $k$ to location $i$, location coordinates to $i$, realization of the continuous function $\beta_{k} \left( {u_{i} ,v_{i} } \right)$ at point $i$, and Gaussian error to location $i$. It is noteworthy that the kernel Fixed Gaussian function was used which highlights the optimal bandwidth found by using the Golden section search with the AIC selection criteria. Also, the Gaussian kernel supported the constant weight, and the value became less from the centre of the kernel but never touched zero. The kernel was suitable for fixed kernel because it could prevent the risk of the absence of data in the kernel. The Fixed Gaussian kernel earlier described [33] is as follows:

2 $$w_{ij} = \text{exp}\left[ { - \left( {d_{ij} /b} \right)^{2} } \right]$$

Also, $w_{ij}$ was the weight value observed at the location $j$ to approximate the calculation of the coefficients on area $i$, $d_{ij}$ was the Euclidean distance between $i$ and $j$, and $b$ was the size of fixed bandwidth given by the size of metric. The Golden section automatically searched the optimal frequency range value by comparing indicators of the model with the bandwidth size. A positive R^2^ indicates a positive correlation. A positive coefficient means X and Y changed in the same direction and if the environmental risk factor increased, then number of confirmed malaria cases increased. Conversely, a negative coefficient means X (explanatory variable) and Y (the response variable) changed in opposite directions. Student’s t distribution that had values outside the range of − 1.97 and 1.97 formed a critical region with a 0.05 (95% CI) level of significance, whereas values outside the range of − 2.59 and 2.59 formed critical regions with a 0.01 (99% CI) level of significance. Step-wise computation performed with these data is shown in Fig. 4.

The locally weighed R^2^ between the observed and fitted values has been calculated to measure how well the model replicates the local malaria incident values around each observation. A variable is correctly clarified for each location by the model if R^2^ = 1 with values ranging from 0 to 1.

To compare the performance between global OLS and local GWR, GWR4 software was also used. We performed an ANOVA testing the null hypothesis that the GWR model represents no improvement over a global model. For local GWR, the sufficient number of degrees of freedom was a function of the bandwidth.

## Results

### Data pre-processing

Multicollinearity does not occur, because the VIF value is less than 10 and the tolerance value is higher than 0.1.

### Environmental factors influencing confirmed malaria cases at global level: OLS model

The global OLS model reveals that altitude and distance to the forest (negative coefficients) and rainfall (positive coefficient) significantly influence the number of malaria cases. Confirmed malaria cases are more common in regions with high rainfall, lowland and areas adjacent to forest. On the other hand, environmental factors such as aspect or direction towards the slope, distance from the river, and the distance from lakes to pond do not have any significant association with malaria cases. Based on OLS model each factor has a different predictor of malaria case preferences in GWR model stage.

### Environmental factors influencing confirmed malaria cases at local level: GWR model

**Fig. 6:**
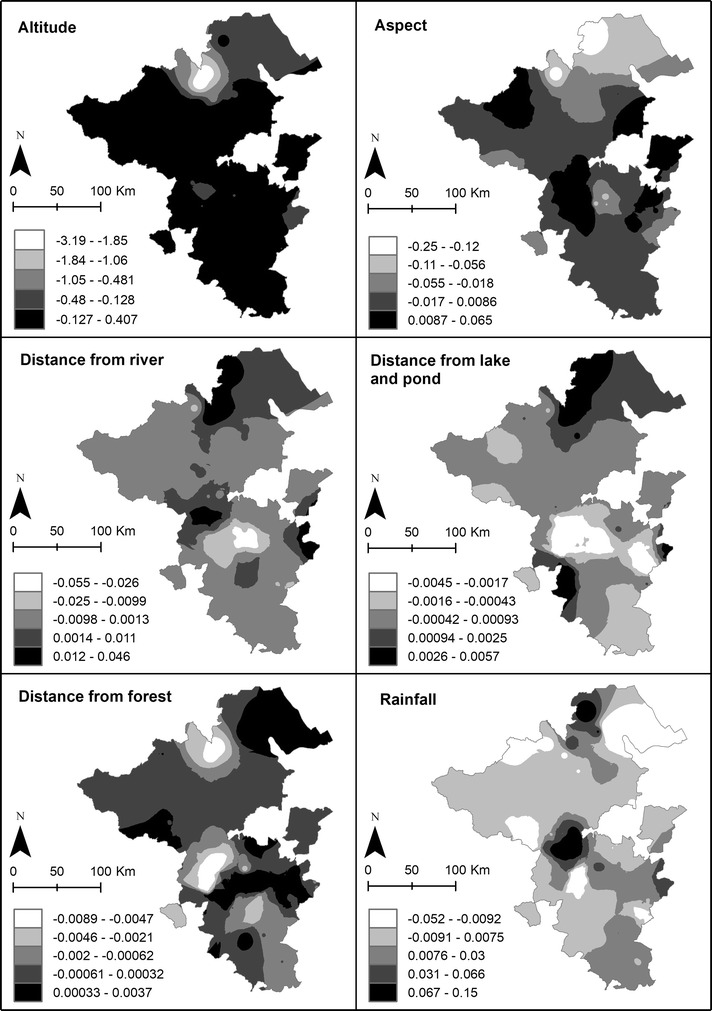
Predicted value from GWR for parameter estimates of explanatory variables of malaria cases in the study area

**Fig. 7:**
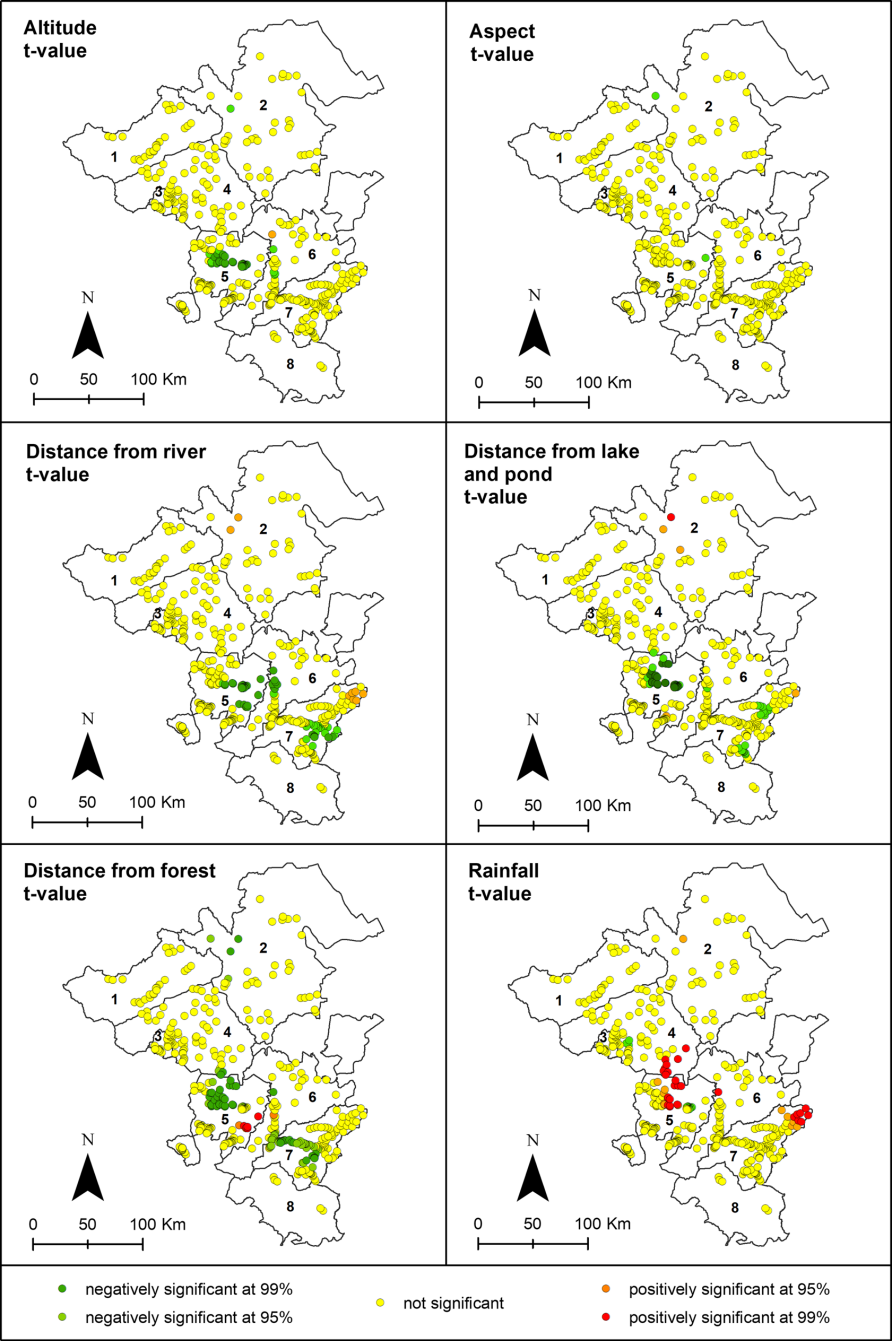
Student’s test significance (95 and 99% confidence interval) for each explanatory variable and village location

**Fig. 8:**
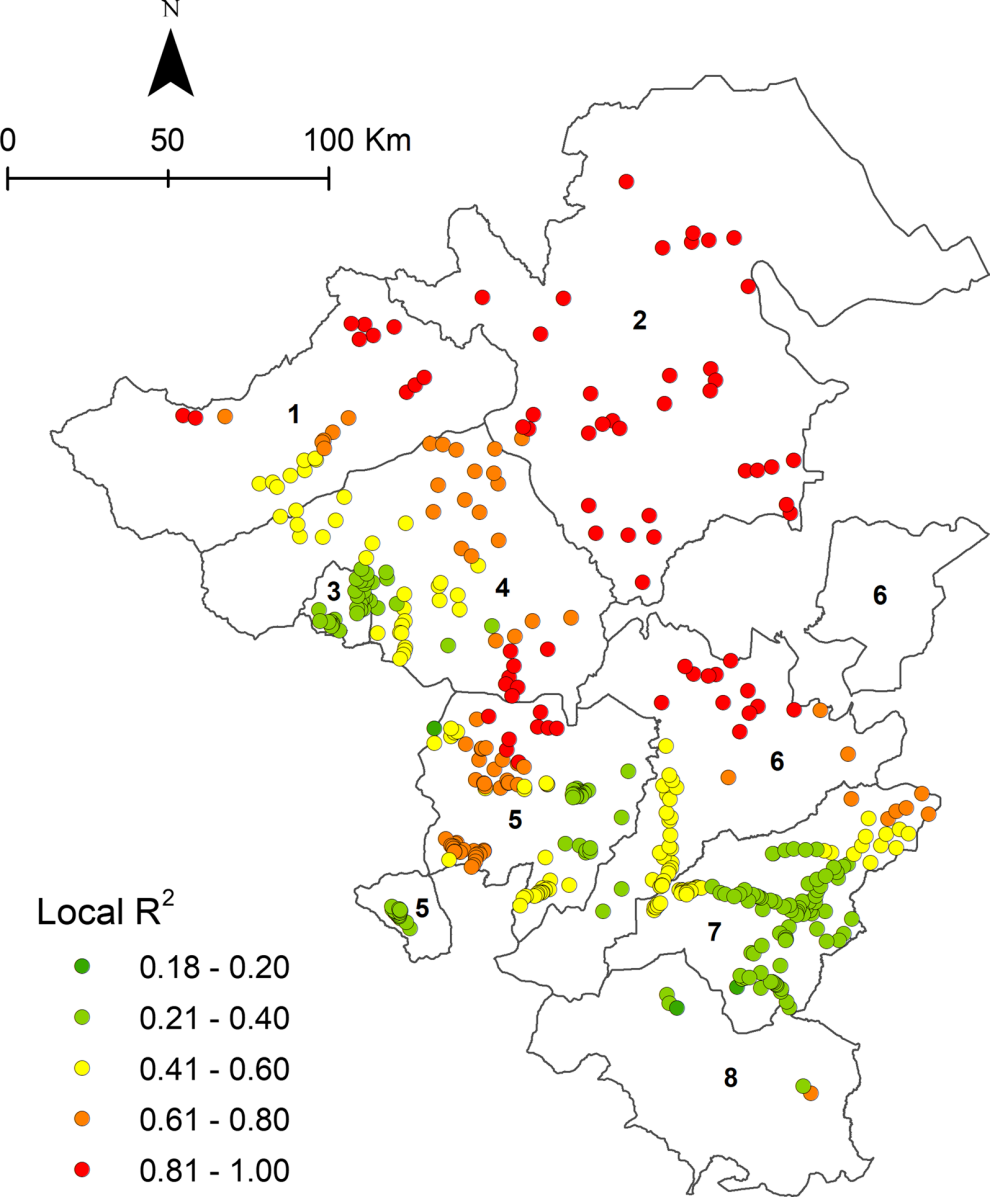
Goodness-of-fit of GWR model (local coefficient of determination R^2^) for malaria cases associated with environmental factors in South Sumatra, Indonesia

The results of GWR using Fixed Gaussian are shown in Table 1. The best bandwidth generates 9184 neighbours and a significant spatial relationship with a specific region has been found. The GWR model provides evidence for a locally different influence of environmental factors on malaria cases as shown by varying parameter estimate value (Fig. 6). “Altitude” and “distance from lake and pond” show a positive association and “aspect” a negative association with malaria incidence in the Northern study area (Musi Banyuasin). “Rainfall” and “distance from river” show a positive association with malaria cases in the Eastern part of Musi Rawas and Lahat. The variables “aspect”, distance from lake and pond” and “distance from forest” are positively associated with confirmed malaria cases in large parts of the study area. The significance thresholds of explanatory variables according to Student’s *t* test in the GWR model are shown in Fig. 7. The local coefficient of determination (local R^2^) for confirmed malaria cases at the local level ranges between 0.18 and 1 (Fig. 8).

**Table 1:** GWR result based on fixed Gaussian (distance) kernel function for geographical weighting

Bandwidth and geographic ranges	Value
Bandwidth size	9184.47
Diagnostic information	
Residual sum of squares	33,549.28
Classic AIC	3482.17
BIC/MDL	4198.30
CV	178.92
R^2^	0.69
Adjusted R^2^	0.41

### Comparison between the two methods OLS and GWR

Like OLS, GWR is a statistical model that provides insights into the relationship between the dependent variable confirmed malaria cases and six independent explanatory variables. GWR is selected as best model based on the residual sum of square, and classic AIC, and the R^2^ as stated in Table 2.

**Table 2:** Comparison between global OLS and local GWR models

Value	OLS	GWR
Residual sum of square	100,625.26	33,549.28
Classic AIC	3625.82	3482.17
R^2^	0.06	0.69
Adjusted R^2^	0.05	0.41

The global regression model indicates that the variables have some influence on the study area (Table 3). The global OLS model explains 6.2% variation of malaria incidences by environmental factors (R^2^ = 0.06). This implies that 93.8% of the malaria incidence is caused by unknown environmental factors related to local variation which are not taken into account in the OLS model [33]. The local GWR explained 68.7% variation in malaria incidences (Y) by environmental factors (R^2^ = 0.69). The DIFF criterion indicates that the spatial distribution of malaria incidence is associated with the independent variables “altitude”, “distance from lakes and pond”, “distance from forest”, and “rainfall” with local spatial heterogeneity (Table 3). Though the testing of local coefficients for “aspect” and “distance from river” suggests no spatial variability (Table 3).

**Table 3:** The result of global regression model and geographical variability test of local coefficients for six environmental factors

Variables	Global regression model output				Geographical variability test			
	Estimate	SE	T value	P value	F	DOF for F test		DIFF of criterion
Intercept	7.98	4.63	1.72	0.04	33.20	10.48	261.38	− 347.99
“Altitude (X1)”	− 0.02	0.00	− 4.03	0.00	0.24	12.02	261.38	19.19
“Aspect (X2)”	− 0.01	0.01	− 1.60	0.05	0.55	22.68	261.38	24.91
“Distance from the river (X3)”	0.00	0.00	− 0.84	0.24	1.84	18.15	261.38	− 16.03
“Distance from lakes and pond (X4)”	0.00	0.00	0.39	0.71	0.90	15.04	261.38	7.99
“Distance from forest (X5)”	0.00	0.00	− 3.69	0.00	2.99	14.61	261.38	− 38.12
“Rainfall (X6)”	0.00	0.00	2.38	0.02	13.07	10.17	261.38	− 158.91

The GWR model explains the relationship between the response variable “confirmed malaria case” and six explanatory variables significantly better than the global regression model OLS (F = 2.12, P < 0.05) (Table 4). The best model weights are automatically determined for each location and are mapped in Fig. 7.

## Discussion

Climate data are frequently used to predict for the spatial, seasonal and interannual variation for malaria transmission, for example the dynamic malaria model forecasting malaria prevalence with seasonal climate published by Hoshen and Morse [35]. The global OLS model revealed here that altitude, distance to forest, and rainfall significantly influence malaria incidence in South Sumatra. Similarly, land use, humidity, altitude and rainfall have been identified by GWR to determine the regional vulnerability to malaria in Purworejo, Indonesia [36]. However, the GWR model considering spatial heterogeneity explains better the association of malaria case with environmental factors in South Sumatra. Likewise in Venezuela, GWR analysis revealed that ecological interactions that act on different scales play a role in malaria transmission and that modelling enhances the understanding of relevant spatiotemporal variability [10]. The environmental factors shown to be significantly associated with malaria cases vary strongly at the village level. This finding is consistent with those obtained in studies in Ethiopia (Addis Ababa), the Amazon region of Brazil (Rondôia), and Cambodia [11, 37, 38]. A validated OLS can lead to a global policy and a validated relationship with GWR is more appropriate to drive to the local system. A geostatistical model based on analysis of residuals and using climatic, population and topographic variables has also been shown to be an important tool for local malaria prediction in Mali [39]. In the highlands of western Kenya, topographic parameters could be used to identify the risk of malaria and thereby helped to improve malaria monitoring or targeted malaria control activities [9].

The relationship of altitude and malaria cases has been shown in present study as well and may relate to the biology of malaria vectors. Globally, *Anopheline* species diversity and density decline from the lowlands to highlands [40]. Accordingly, poor villagers living in forested lowland areas in Papua, Indonesia, were found to be at higher risk of malaria infection than those in the highlands [41]. In contrast, a positive correlation between altitude and the abundance of *Anopheles* mosquitoes has observed in the highlands of Ethiopia, Colombia and Ecuador, particularly in warmer years [42–44]. This observation may be related to the direction towards the slopes as the distribution and density of mosquito populations may be affected by wind direction [45]. In an Ethiopian study, minimum temperatures were significantly associated with malaria cases in cold areas, while precipitation was associated with transmission in hot areas [46]. In accordance to many studies, malaria case was significantly associated with rainfall in villages of South Sumatra. Rainfall showed correlation with the incidence of clinical malaria cases in Tubu village, Botswana [47]. Variations in monthly rainfall in rural Tanzania were largely associated with malaria [48]. Rainfall creates oviposition sites for female mosquitoes, whereas humidity is a key parameter for adult mosquito daily survival [49]. *Anopheline* mosquitoes require stagnant water to complete their larval and pupal development. Thus, rainfall affects the transmission of malaria by providing water to create aquatic habitats. The number of malaria cases was significantly positively connected with higher winter rainfall, but also with a higher average maximum temperature and significantly negatively associated with increasing distance from water bodies in South Africa [50]. Southern Africa Development Community estimates the positive correlation between increasing rainfall and the number of cases in Botswana during 2013 and 2014 [51].

Next to climatic and environmental factors, distance of houses to a forest are interrelated through anthropogenic activities influencing the local and regional climate [52, 53]. These observations can be confirmed for the relationship of malaria case with distance to lake, pond and forest for South Sumatra. A cross-sectional view in Brazil revealed for example that malaria incidence across health districts is positively correlated with the percentage of aggregated deforestation  [26]. Indonesia contributes indeed significantly to deforestation in Southeast Asia. *Anopheles* was reported from eight sources at 47 independent sites. The first record of *Anopheles parangensis* from Sumatra was reported by O’Connor and Sopa (1981), but with no details on location [54]. *Anopheles (Cellia) leucosphyrus* is considered to be of epidemiological importance for malaria transmission in forested areas of Sumatra [54]. In current research, the main *Anopheles* vector diversity in each study area was however not investigated.

**Table 4:** ANOVA testing the null hypothesis that the GWR model represents no improvement over a global model

Source	SS	DF	MS	F Count	F Table
Global residuals	100,625.26	429.00			
GWR improvement	67,075.98	197.74	339.22		
GWR residuals	33,549.28	231.26	145.07	2.34	2.12

Present study has identified Lahat as the South Sumatran district in which environmental factors were of greatest relevance for malaria incidence. Lahat District has both lowland and mountain regions and is home to diverse ethnic groups, such as the Gumai who live along the rivers of the highland areas [55].

One of the key activities for malaria elimination should be the establishment of systems and tools to reduce disease burden where local transmission is high. By comparing the local GWR model with the global OLS model (Table 4), it became apparent that GWR yielded new information about the spatial variation of malaria incidence and thereby better explains local phenomena. The variability of malaria cases in our study was due to environmental and geographical local differences [8]. GWR should be used as a diagnostic model discovering spatially varying relationships between confirmed malaria cases and environmental factors. The use of GWR allows the uncovering of significant environmental variation for malaria incidence, which has previously been unobservable in a specific location [56].

### Limitations of research

Due to practical constraints, this study was unable to encompass the entirety of environmental factors, particularly climate parameters, temperature and humidity, for which only limited data were available and hence not-representative data could not be included. Also the factor land use was eliminated. Malaria location information was plotted using a village centre approach which ignored all other locations where actual infections may have occurred (e.g., forests, plantations). The number of positive malaria per village, did not include the specific coordinates of each positive malaria case and thus, each positive case was placed in the centre of the settlement. Therefore, if land use variables would be involved, there will very likely be a strong bias. However, these eliminated or uninvestigated variables may be correlated with existing variables, for example, the temperature connected with altitude and with aspect or direction of the slope. In the same way, land use may be associated with the distance from the river and the distance from lakes and ponds. Thus, although these parameters (temperature, humidity, land use) were excluded from analysis, these environmental factors were represented by our chosen set of variables. In the future, additional explanatory variables should be addressed to provide a comprehensive review of malaria in the study area. It should comprise, for example, the behavior of mosquito vectors and that of community members, the access to and the delivery of health services, and other eco-bio-social factors that affect the incidence of malaria. Despite these limitations, our study sheds light on relevant information, not only in regional but also local realities regarding environmental variation which might interplay with vector-host relationships and sociocultural practice and provide a suitable environment for malaria mosquitoes.

## Conclusion

In the present study, the importance of different environmental and geographic parameters for malaria disease was shown at global and village-level in South Sumatra, Indonesia. The independent variables altitude, distance from forest, and rainfall in global OLS were significantly associated with malaria cases. As shown by GWR model and in line with recent reviews, the relationship between malaria and environmental factors in South Sumatra was found to vary spatially in different regions. A more in-depth understanding of local ecological factors influencing confirmed malaria case cannot only be used for developing sustainable regional malaria control programs but can also benefit malaria elimination efforts at village level.
